# Neuronal intranuclear inclusion disease with subclinical peripheral neuropathy: A case report

**DOI:** 10.1097/MD.0000000000040636

**Published:** 2024-11-22

**Authors:** Lu Sun, Lihua Zhou, Liyan Ren, Chunru Han, Qun Xue, Linqing Ma

**Affiliations:** a Department of Neurology, The People’s Hospital of Suzhou New District, Suzhou, Jiangsu, China; b Department of Neurology, The First Affiliated Hospital of Soochow University, Suzhou, Jiangsu, China.

**Keywords:** diffusion-weighted imaging, neuronal intranuclear inclusion disease, *NOTCH2NLC* gene, peripheral nerve, skin biopsy

## Abstract

**Rationale:**

Neuronal intranuclear inclusion disease (NIID) is a slowly progressing neurodegenerative disease with various manifestations and high heterogeneity. Clinical characteristics, imaging, skin biopsy, and genetic testing are necessary for its diagnosis. Electromyography may also be a useful tool for diagnosing NIID. In this study, we report a patient with motor and sensory nerve demyelination changes accompanied by axonal damage.

**Patient concerns:**

A 64-year-old woman was admitted to our department with gradually worsening forgetfulness for over a year. After 6 years of follow-up, the symptoms progressively deteriorated.

**Diagnoses:**

Cerebrospinal fluid analysis revealed increased protein levels. Brain magnetic resonance imaging showed characteristic “ribbon-like” high signals in the corticomedullary junction area on diffusion-weighted imaging. High-intensity signals in the white matter were also observed on T2 and fluid-attenuated inversion recovery imaging. Electromyography revealed multiple peripheral nerve damage and conduction changes, including motor and sensory nerve demyelination changes, accompanied by axonal damage. Skin biopsy revealed inclusion bodies with strong positive staining for P62 and ubiquitin antibodies in the nuclei of sweat gland cells, adipocytes, and fibroblasts. Genetic testing indicated that the number of GGC repeats in *NOTCH2NLC* alleles were 14 and 134, respectively. Consequently, the patient was diagnosed with NIID.

**Interventions:**

Currently, no effective treatment is available to delay the progression of the disease.

**Lessons:**

We report a case of NIID with subclinical peripheral neuropathy, although the patient did not experience sensory symptoms such as numbness in the extremities. Electromyography can be used to detect subclinical peripheral nerve damage.

## 1. Introduction

Neuronal intranuclear inclusion disease (NIID) is a slowly progressive neurodegenerative disease characterized by eosinophilic intranuclear inclusions in various tissue cells.^[1]^ NIID was first reported by Lindenberg et al in 1968.^[2]^ In 2011, Sone et al discovered eosinophilic intranuclear inclusions in the skin tissue of patients with NIID.^[3]^ Since then, the rates of diagnosis of patients with NIID have increased significantly. Initially, the primary methods for diagnosing this disease were abnormally high signal intensity in the corticomedullary junction on diffusion-weighted magnetic resonance imaging (MRI) and characteristic findings on skin biopsy.^[4,5]^ In 2019, a GGC repeat expansion mutation in the untranslated region of the notch 2 N-terminal like C (*NOTCH2NLC*) gene was identified as the causative agent of NIID,^[6–9]^ providing crucial evidence for the diagnosis of the disease.

NIID exhibits diverse and highly heterogeneous manifestations. Clinical manifestations include cerebellar ataxia, Parkinson syndrome, peripheral neuropathy, autonomic nervous system dysfunction, dementia, and retinopathy.^[4]^ Notably, NIID is often misdiagnosed or overlooked in clinical practice. Dementia is the initial core symptom of NIID in adults. We herein describe the case of a woman with NIID presenting with cognitive impairment as the primary clinical feature and accompanied by subclinical peripheral nerve damage. We summarize the clinical characteristics, imaging findings, skin pathological changes, and genetic testing results of the patient. In addition, we review the relevant literature to provide insight into the diagnosis of patients with NIID.

## 2. Case presentation

A 64-year-old woman was admitted to our department in 2018 with a year-long history of gradually worsening forgetfulness, particularly affecting her short-term memory. Over this period, the patient progressively lost the ability to perform household tasks such as cooking or buying groceries, although she was still able to take care of herself. The symptoms gradually deteriorated, and were accompanied by dizziness, frequent urination, difficulty in falling asleep, and poor sleep quality. However, the patient did not exhibit any obvious limb weakness or numbness. In addition, the patient did not experience any convulsions or difficulty in swallowing. The patient had a history of diabetes but denied any history of drug abuse or exposure to toxins. Furthermore, the patient had no family history of genetic diseases.

The patient appeared mentally alert, spoke fluently, and was cooperative during the physical examination. However, the memory, calculation, and orientation abilities were noticeably reduced. Both pupils were round (approximately 2.0 mm) and responsive to light reflection. Muscle strength in the limbs was normal, and sensations in the face, trunk, and extremities were intact. The bilateral tendon reflexes were symmetrical and normal, while the pathological reflexes were negative on both sides. The Mini-Mental State Examination score was 16/30.

Laboratory test results, including routine blood tests, C-reactive protein, routine urine, routine stool, coagulation function, blood lipids, liver function, renal function, tumor markers, human immunodeficiency virus antibodies, anti-treponema pallidum-specific antibodies, hepatitis B antigen antibodies, hepatitis C antibodies, glycosylated hemoglobin, thyroid function, immune series, phospholipid antibodies, and erythrocyte sedimentation rate, were within normal ranges. The blood glucose and homocysteine levels were 6.20 mmol/L and 16.60 μmol/L, respectively.

**Figure 1.**
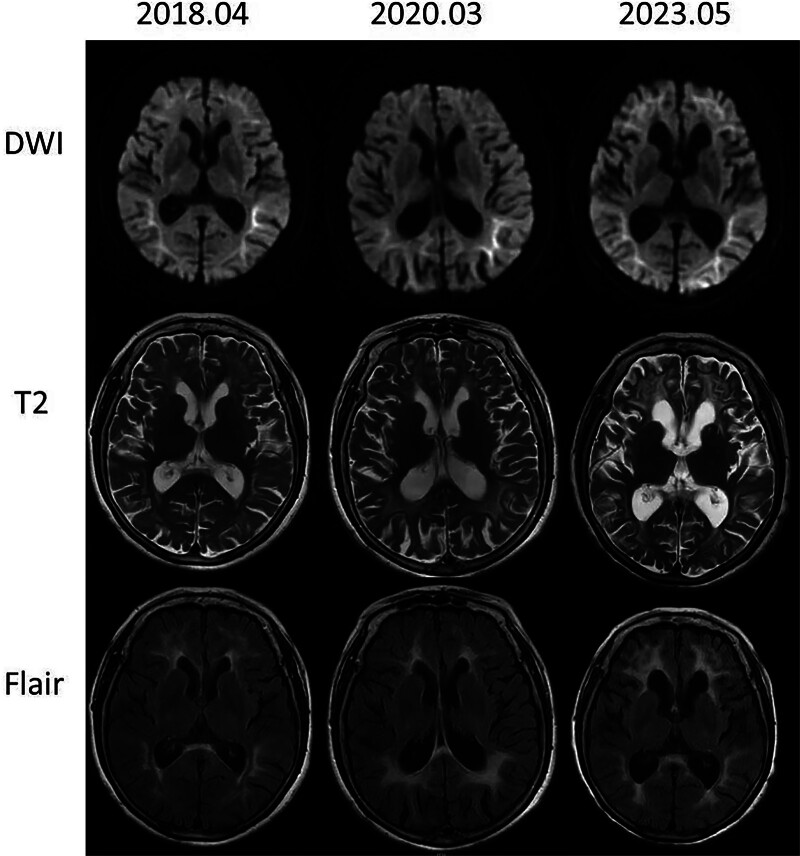
Diffusion-weighted imaging (DWI) showing subcortical hyperintense signals along the junction area of the cortex and white matter. T2 and fluid-attenuated inversion recovery (FLAIR) images exhibiting progressive diffuse confluent white matter changes.

Lumbar puncture revealed a pressure of 70 mm H_2_O. Cerebrospinal fluid analysis revealed a white blood cell count of 1 × 10^6^/L, protein level of 969 mg/L, chlorine level of 129 mmol/L, and glucose level of 6.51 mmol/L. MRI revealed abnormally high signals in the corticomedullary junction area on diffusion-weighted imaging (DWI) and fluid-attenuated inversion recovery (FLAIR) images. These lesions gradually expanded with disease progression (Fig. 1). An electromyographic nerve conduction velocity test demonstrated conduction changes indicative of nerve damage, including motor and sensory nerve demyelination changes accompanied by axonal damage (Table 1). Electroencephalography results were normal.

**Table 1:** Results of nerve conduction studies.

Motor nerves		DL (ms)/normal value	CMAP (mv)/normal value	MCV (m/s)/normal value	F (ms)/normal value
Median nerve	Left	4.10/≤4.2	4.7/≥4.8	37.1/≥50.0	34.9/≤27.2
	Right	4.75/≤4.2	4.7/≥4.8	38.0/≥50.0	35.9/≤27.2
Ulnar nerve	Left	2.78/≤3.1	5.0/≥5.5	42.2/≥51.0	35.9/≤28.4
	Right	2.68/≤3.1	6.9/≥5.5	41.3/≥51.0	35.6/≤28.4
Tibial nerve	Right	7.00/≤5.8	3.8/≥5.0	37.0/≥39.4	64.0/≤55.0
Peroneal nerve	Right	3.90/≤4.6	0.57/≥2.3	31.1/≥39.8	60.4/≤55.0

Sensory nerves		DL (ms)	CNAP (mv)/nomal value	SCV (m/s)/nomal value	
Median nerve	Left	2.98	6.9/≥8.0	40.3/≥50.8	
	Right	2.83	4.7/≥8.0	40.6/≥50.8	
Ulnar nerve	Left	NE	NE/≥5.0	NE/≥50.6	
	Right	2.66	1.73/≥5.0	40.2/≥50.6	
Sural nerve	Right	2.22	5.6/≥7.0	36.0/≥41.9	
Superficial peroneal nerve	Right	2.53	2.2/≥4.0	33.6/≥41.3	

CMAP = compound muscle action potential, DL = distal latency, F = F-latency, MCV = motor nerve conduction velocity, NE = not evoked, SCV = sensory nerve conduction velocity, SNAP = sensory nerve action potential.

**Figure 2.**
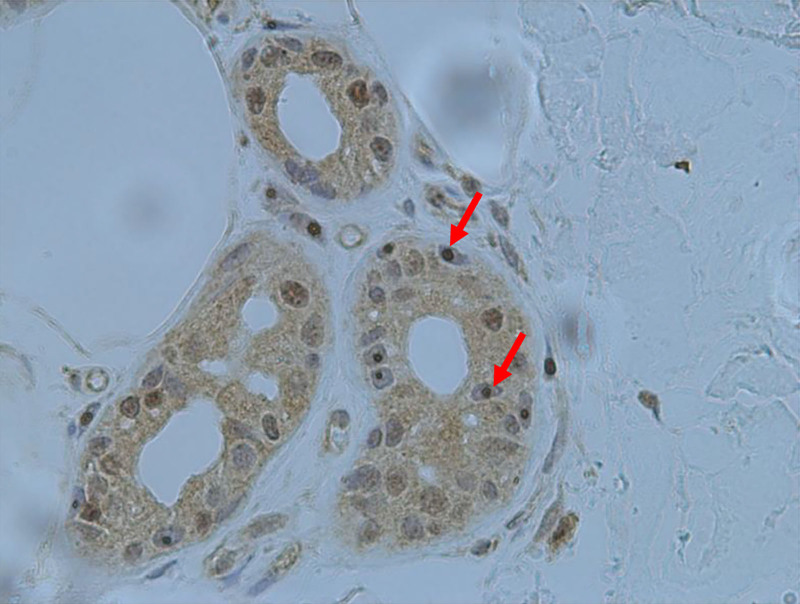
Immunohistochemistry of the skin biopsy revealed inclusion bodies in the nuclei of some sweat gland cells, adipocytes, and fibroblasts, with strong positive staining for P62 and ubiquitin antibodies. Inclusion bodies are indicated with arrows (immunohistochemistry was performed by the pathology laboratory of the Department of Neurology, Beijing Tiantan Hospital, Capital Medical University).

The skin biopsy site was located approximately 10 cm above the right lateral malleolus. A tissue sample measuring approximately 1.5 cm × 1.0 cm, including epidermis, dermis, and subcutaneous tissue, was removed and fixed in 10 % formalin. Immunohistochemistry revealed inclusion bodies in the nuclei of some sweat gland cells, adipocytes, and fibroblasts, with strong positive staining for P62 and ubiquitin antibodies (Fig. 2).

Genetic testing revealed that the number of GGC repeats in the *NOTCH2NLC* gene (associated to NIID pathology) were 14 and 134, respectively. The number of repeats on 1 allele exceeded the normal range (<38 repeats). Notably, a number of repeats ≥ 66 is considered pathogenic. In contrast, the number of GGC repeats were 14 and 23 in the *NOTCH2NLC* gene from her son, who remained asymptomatic.

The patient has been followed up for 6 years, during which the symptoms gradually worsened. The patient became bedridden after an accidental fall 3 years after discharge and was unable to care for herself. The patient also stopped being able to recognize her family and express discomfort properly. Distal muscle atrophy was observed. The bilateral tendon reflexes were decreased. Additional nerve conduction studies showed worsening of motor and sensory nerve demyelination, and axonal damages (Table 2). Unfortunately, needle electromyography was not performed due to refusal from the family.

**Table 2:** Results of follow-up nerve conduction studies.

Motor nerves		DL (ms)/normal value	CMAP (mv)/normal value	MCV (m/s)/normal value	F (ms)/normal value
Median nerve	Left	4.42/≤4.2	4.8/≥4.8	37.9/≥50.0	28.1/≤27.2
	Right	5.07/≤4.2	3.4/≥4.8	37.2/≥50.0	NE/≤27.2
Ulnar nerve	Left	4.45/≤3.1	0.91/≥5.5	30.7/≥51.0	30.3/≤28.4
	Right	8.73/≤3.1	0.13/≥5.5	39.4/≥51.0	31.1/≤28.4
Tibial nerve	Right	9.21/≤5.8	0.27/≥5.0	22.6/≥39.4	NE/≤55.0
Peroneal nerve	Right	NE/≤4.6	NE/≥2.3	NE/≥39.8	NE/≤55.0

Sensory nerves		DL (ms)	CNAP (mv)/nomal value	SCV (m/s)/nomal value	
Median nerve	Left	3.1	3.3/≥8.0	37.1/≥50.8	
	Right	3.07	2.2/≥8.0	39.1/≥50.8	
Ulnar nerve	Left	NE	NE/≥5.0	NE/≥50.6	
	Right	2.86	4.6/≥5.0	38.5/≥50.6	
Sural nerve	Right	3.56	1.05/≥7.0	36.5/≥41.9	
Superficial peroneal nerve	Right	NE	NE/≥4.0	NE/≥41.3	

CMAP = compound muscle action potential, DL = distal latency, F = F-latency, MCV = motor nerve conduction velocity, NE = not evoked, SCV = sensory nerve conduction velocity, SNAP = sensory nerve action potential.

## 3. Discussion

NIID was first reported in 1968 in a 28-year-old man.^[2]^ The patient began to develop progressive rigidity and ataxia during childhood and experienced delays in intellectual and behavioral development. The patient eventually died of complications. Eosinophilic inclusions were reported to be widely distributed in the brain and internal organs. In addition, intranuclear inclusions have also been observed in rectal, gastrointestinal, sural nerve, and renal biopsy specimens. Sone et al performed skin biopsy in patients with adult-onset NIID and found characteristic intranuclear eosinophilic inclusions in adipocytes, fibroblasts, and sweat gland cells. Moreover, staining for ubiquitin and SUMO-1 were positive. Hence, skin biopsy has emerged as a useful tool for diagnosing NIID.^[3]^ Its relatively noninvasive nature has increased the diagnostic yield of NIID.

The disease is classified as pediatric, juvenile, or adult NIID based on the age of the patient at onset.^[2]^ The pediatric form manifests before 5 years of age, with a relatively rapid progression and a course lasting < 10 years. The clinical manifestations of patients with the juvenile form are more heterogeneous, and the survival period after onset is usually 10 to 20 years.^[10]^ Most patients with adult-onset NIID develop the disease after 50 years of age. Dementia is the most common clinical manifestation of sporadic adult-onset NIID. Several patients may have core stenoma. Other symptoms of autonomic nervous system (ANS) involvement include bladder dysfunction^[11]^ and vomiting.^[12]^ Patients may also exhibit ataxia^[13]^ and peripheral nerve damage, including myasthenia^[14]^ and sensory impairment. Other clinical features include tremors, rigidity, abnormal behavior, and convulsions.^[12]^ The patient in the present case was initially diagnosed with progressive dementia. Although the patient did not have sensory symptoms such as numbness of the extremities, electromyography showed peripheral nerve damage, indicating subclinical peripheral neuropathy. Currently, no effective treatments are available for this condition.

MRI is crucial for identifying NIID. In the DWI sequence, persistent abnormal high-signal lesions are distributed along the junction area of the cortex and white matter, exhibiting characteristic flame-like shapes such as curve-, cockscomb-, and ribbon-like patterns.^[15,16]^ In the early stages of the disease, these lesions are primarily limited to the frontal lobe. However, as the disease progresses, high signals gradually develop in other areas while sparing the deep white matter.^[1]^ Most patients exhibit bilateral symmetrical and diffuse white matter lesions on T2 and FLAIR images. The frontal lobe is more commonly affected, with possible involvement of the external capsule. Some experts have suggested that frontal lobe leukoencephalopathy could serve as a sensitive and early diagnostic indicator.^[17]^ In the present case, DWI sequences revealed characteristic ribbon-like hypersignals, whereas T2 and FLAIR sequences showed symmetrical leukoencephalopathy. The high-intensity signals observed on DWI, FLAIR, and T2 gradually expanded with disease progression, in consistency with the findings of previous studies.^[18]^

Recently, the abnormal expansion of GGC repeats in *NOTCH2NLC* was established as a factor in NIID pathogenesis.^[6–9]^ Patients with NIID presenting with cognitive impairment, have approximately 120 GGC repeats. In the present case, 134 GGC repeats were detected in the gene sequence, consistent with the genetic characteristics of patients with NIID whose primary manifestation is cognitive impairment.

However, the diagnosis of NIID remains challenging in certain patients. First, NIID presents significant clinical heterogeneity and covers a broad range of differential diagnoses, especially when patients exhibit symptoms associated only with a single target organ. This variability can lead to clinical misdiagnoses or missed diagnoses. Second, previous research has shown that *NOTCH2NLC* is associated with other neurodegenerative diseases, including Alzheimer,^[19,20]^ frontotemporal dementia,^[20]^ Parkinson,^[19]^ adult white matter lesions,^[21]^ essential tremor^[22]^ and multiple system atrophy.^[23]^ Intranuclear eosinophilic inclusions have been detected in biopsies collected from patients with these conditions. Moreover, imaging findings may appear normal in patients with early-stage NIID, further complicating the differentiation between NIID and the previously mentioned neurodegenerative diseases, all of which exhibit eosinophilic intranuclear inclusions and abnormal number of GGC repeats in *NOTCH2NLC*. Therefore, accurately distinguishing NIID from other diseases is crucial.

The NIID diagnostic rate has significantly improved owing to advances in imaging techniques, the widespread use of skin biopsy pathology, and notably, the identification of pathogenic genes. Electromyography can be used to detect subclinical peripheral nerve damage in NIID. The GGC repeat expansion in *NOTCH2NLC* has been reported to be correlated with peripheral neuropathy.^[24]^ However, our study had some limitations. Despite the detection of electrophysiological abnormalities in the present case, its diagnostic value for patients with NIID remains unclear. The findings of this study not only enhance our understanding of NIID but also present additional challenges that need to be addressed. Further research is necessary to investigate the fundamental mechanisms underlying the occurrence and progression of NIID for facilitating improved clinical diagnosis and the development of effective treatment strategies.

## 4. Conclusion

In summary, we presented the case of a patient with NIID. The diagnosis of NIID was confirmed by combining neuroimaging characteristics, skin biopsy findings, and genetic analysis results. Notably, in the present case, abnormal electrophysiological findings revealed peripheral neuropathy, suggesting that electromyography may be used as an assistive method in NIID diagnosis.

## Acknowledgments

We thank the patient and her family for their participation in the study. We also thank Editage for English language editing.

## Author contributions

**Conceptualization:** Lu Sun, Linqing Ma.

**Data curation:** Lu Sun.

**Formal analysis:** Lihua Zhou.

**Funding acquisition:** Linqing Ma.

**Methodology:** Liyan Ren.

**Resources:** Chunru Han.

**Supervision:** Qun Xue, Linqing Ma.

**Writing – original draft:** Lu Sun.
